# High Rates of Detection of Respiratory Viruses in Tonsillar Tissues from Children with Chronic Adenotonsillar Disease

**DOI:** 10.1371/journal.pone.0042136

**Published:** 2012-08-03

**Authors:** Jose Luiz Proenca-Modena, Fabiana Cardoso Pereira Valera, Marcos Gerhardinger Jacob, Guilherme Pietrucci Buzatto, Tamara Honorato Saturno, Lucia Lopes, Jamila Mendonça Souza, Flavia Escremim Paula, Maria Lucia Silva, Lucas Rodrigues Carenzi, Edwin Tamashiro, Eurico Arruda, Wilma Terezinha Anselmo-Lima

**Affiliations:** 1 Department of Cell Biology, School of Medicine of Ribeirao Preto of University of São Paulo, Ribeirao Preto, Brazil; 2 Department of Ophthalmology, Otorhinolaryngology and Head and Neck Surgery, School of Medicine of Ribeirao Preto of University of São Paulo, Ribeirao Preto, Brazil; 3 Virology Research Center, School of Medicine of Ribeirao Preto of University of São Paulo, Ribeirao Preto, Brazil; University of Liverpool, United Kingdom

## Abstract

Chronic tonsillar diseases are an important health problem, leading to large numbers of surgical procedures worldwide. Little is known about pathogenesis of these diseases. In order to investigate the role of respiratory viruses in chronic adenotonsillar diseases, we developed a cross-sectional study to determine the rates of viral detections of common respiratory viruses detected by TaqMan real time PCR (qPCR) in nasopharyngeal secretions, tonsillar tissues and peripheral blood from 121 children with chronic tonsillar diseases, without symptoms of acute respiratory infections. At least one respiratory virus was detected in 97.5% of patients. The viral co-infection rate was 69.5%. The most frequently detected viruses were human adenovirus in 47.1%, human enterovirus in 40.5%, human rhinovirus in 38%, human bocavirus in 29.8%, human metapneumovirus in 17.4% and human respiratory syncytial virus in 15.7%. Results of qPCR varied widely between sample sites: human adenovirus, human bocavirus and human enterovirus were predominantly detected in tissues, while human rhinovirus was more frequently detected in secretions. Rates of virus detection were remarkably high in tonsil tissues: over 85% in adenoids and close to 70% in palatine tonsils. In addition, overall virus detection rates were higher in more hypertrophic than in smaller adenoids (p = 0.05), and in the particular case of human enteroviruses, they were detected more frequently (p = 0.05) in larger palatine tonsils than in smaller ones. While persistence/latency of DNA viruses in tonsillar tissues has been documented, such is not the case of RNA viruses. Respiratory viruses are highly prevalent in adenoids and palatine tonsils of patients with chronic tonsillar diseases, and persistence of these viruses in tonsils may stimulate chronic inflammation and play a role in the pathogenesis of these diseases.

## Introduction

Palatine tonsils and adenoids are prominent secondary lymphoepithelial organs associated with the upper respiratory tract, where inhaled antigens first come in contact with host defense cells [1]. Tonsillar hyperplasia and recurrent tonsillitis are common chronic diseases that often lead to complications that include nasal obstruction, recurrent sinusitis, snoring, auditory tube dysfunction, otitis media, obstructive sleep apnea and changes in facial growth and behavioral development [2]. These complications frequently lead to patients undergoing tonsillectomy, which still is one of the most commonly performed pediatric surgical procedures worldwide [3]. Yet, the pathogenesis of chronic tonsillar diseases is largely unknown. Tonsils and adenoids harbor bacterial and viral pathogens [4]–[6], and although bacterial components of biofilms can be cultured from the surfaces and crypts of inflamed tonsils and adenoids, their role as causative agents of adenotonsillar hyperplasia is largely unknown [7].

Recovery of adenovirus and herpesvirus from tonsils and adenoids has been reported since the 1950s and 1960s [8], [9], but the advent of sensitive molecular methods has made it possible to detect many other viruses, including those not amenable to recovery in cell culture [4], [5], [10] **.** Nevertheless, systematic studies of viruses in adenoids and tonsils have been scarce, especially in association with chronic hypertrophic tonsillar disease. In order to investigate the role of respiratory viruses in chronic adenotonsillar diseases we have performed a cross-sectional study in surgical tissue samples and cavity washes from patients with recurrent tonsillitis and chronic adenotonsillar hyperplasia. To the best of our knowledge, this has been the most extensive study of its kind to date. The results revealed high rates of virus detection in adenotonsillar tissues, with divergent virus preponderance in different sampling sites, possibly owing to different routes of infection and/or tissue composition. The high rates of detection of respiratory viruses in adenoids and palatine tonsils of patients with chronic adenotonsillar disease bring new insights about the transmission of respiratory viruses and their role in pathogenesis of chronic adenotonsillar diseases.

## Materials and Methods

### Patients and Specimen Collections

A total of 121 children (63 boys) 1 to 14 years of age (mean age 5.9±3.2 years years) were enrolled in the study, all with indication for tonsillectomy due to tonsillar hypertrophy (60) or recurring tonsillitis (61), and in the absence of symptoms of acute respiratory infection.

This cross-sectional study was carried out from May 2010 to August 2011 and it enrolled patients who underwent tonsillectomy at the Division of Otorhinolaryngology of the Clinical Hospital of the University of São Paulo School of Medicine, in Ribeirão Preto, Brazil. Exclusion criteria were: presence of symptoms of acute respiratory infection at the time of surgery and antibiotic treatment within one month prior to surgery.

Patients were grouped according to palatine tonsil size and history of prior tonsillar infections as follows:

RT group: Included 11 patients (9.1%) with 4 to 14 years of age (mean age 5.91 years) with recurrent tonsillitis (RT) according to the Paradise criteria: 7 episodes in the last year, 5 episodes/year in the last two years, or 3 episodes/year in the last 3 years; plus tonsils that occupied less than 50% of the oropharyngeal airway (Brodsky tonsil size ≤2).
TH group: Included 49 patients (40.5%) with 2 to 13 years of age (mean age 5.85 years) with only tonsillar hyperplasia (TH) with enlarged tonsils occupying 50–100% of the oropharyngeal airway (Brodsky tonsil size ≥2), without history of recurrent tonsillitis.
RT/TH group: Included 50 patients (41.3%) with 1 to 14 years of age (mean 6.33 years) with recurrent tonsillitis and tonsillar hyperplasia (RT/TH).
Non RT/TH group: Included 11 patients (9.1%) with 3 to 10 years of age (mean of 7.09 years), with normal size tonsils and no history of recurrent tonsillitis. These patients presented tonsils that occupied less than 50% of the oropharyngeal airway (Brodsky tonsil size ≤2), but they had OSAS - obstructive sleep apnea syndrome.

According to adenoid size, as determined by lateral X-ray and/or nasal endoscopy, the 119 patients who underwent adenoidectomy (2 patients who underwent tonsillectomy did not have adenoids removed), were grouped as follows:

Group 1: Included 15 patients (12.6%) with 3 to 13 years of age (mean of 7 years), with less than 50% airway obstruction.
Group 2: Included 58 patients (48.7%) with 1 to 14 years of age (mean of 6.3 years) with 50–75% airway obstruction.
Group 3: Included 46 patients (38.7%) with 2 to 14 years of age (mean 5.3 years), with airway obstruction greater than 75%.

Samples obtained during surgery were: a) nasopharyngeal secretions (NS), consisting of a combination of nasopharyngeal washes with swabs from mucosal surfaces of adenoids and palatine tonsils, all three collected from all patients; b) fragment of tissue from removed adenoids and palatine tonsils; c) peripheral blood. Tissue fragments were placed in Eagle’s minimal essential medium (MEM) with 10% fetal bovine serum and 15% antibiotics-antimycotic solution (penicillin-streptomycin 20,000 U/ml and amphotericin B 200 µg/mL) (Gibco, Grand Island, NY, USA) and all samples were transported on ice within less than four hours to the virology laboratory, where they were processed and tested for the respiratory viruses.

In the laboratory, the tissue fragments were washed twice with MEM to remove excess of cells debris and blood, and cut into portions of approximately 0.5 cm x 0.5 cm using RNAse free surgical material. These fragments were macerated in Trizol (Invitrogen, Carlsbad, CA, USA) and in one preservative solution (RNA later – Invitrogen, Carlsbad, CA, USA) for further nucleic acids extraction. All the secretions were treated with antibiotics and antimycotics (Gibco, Grand Island, NY, USA) for 1 hour and aliquoted in an equal volume of freezing medium (MEM, 20% FBS and 15% glycerol) and in the triple volume of Trizol (Invitrogen, Carlsbad, CA, USA).

After processing, all samples were aliquoted and kept at −70°C until further testing.

### Ethics Statement

The project was based on analysis of clinical samples from patients sent for tonsillectomy, and all obtained tissues would normally excised from all patients. The study was conducted according to the principles expressed in the Declaration of Helsinki and was approved by the Ethics Review Committee of the Hospital das Clínicas de Ribeirão Preto (Number 10466/2008) and a written informed consent was obtained from all parents and guardians prior to enrollment.

### Detection of Respiratory Viruses

Nucleic acids were extracted from 200 µL of nasopharyngeal secretions using the QIAamp Min Elute Virus Spin Kit (Qiagen GmbH, Hilden, Germany); from ∼30 mg of tissue using the AllPrep DNA/RNA mini kit (Qiagen GmbH, Hilden, Germany); and from 1 mL of peripheral blood using the QIAamp RNA and DNA blood mini kit (Qiagen GmbH, Hilden, Germany), all following manufacturer’s instructions.

Samples were tested by TaqMan real-time PCR (qPCR) for the presence of genomes of human bocavirus (HBoV), human adenovirus (HAdV), rhinovirus (HRV), enterovirus (HEV), human respiratory syncytial virus (HRSV) A and B, metapneumovirus (HMPV) A and B, human Influenza virus (FLU) A and B (including H1N1 2009), parainfluenza virus (HPIV) 1 and 3, and coronavirus (HCoV) OC43 and 229E. All qPCR assays were done on Thermocycler 7300 (Applied Biosystems, Foster City, CA, USA) with specific primers and probes (Table S1 **)**. qPCR for β-actin and RNAseP housekeeping genes was done in all samples. The qPCR assays using two sets primers for different clusters of HRV types, HRSV subgroups A and B, and HCoV types OC43 and 229E, were done in duplex format. The remaining qPCR assays done with one set of primers per reaction for HAdV, HBoV, HMPV A, HMPV B, FLU A, FLU B, HPIV 1, HPIV 3, RNAseP and β-Actin were done separately. For the RNA viruses (HRV, FLU, HPIV, HRSV, HMPV, HEV and HCoV) reverse transcription was done prior to qPCR using 1 µg of extracted RNA primed with random hexamers, with Multiscribe reverse transcriptase (Applied Biosystems, Foster City, CA, USA) following protocol proposed by the manufacturer. Duplex qPCR assays were done in a final volume of 15 µL with 3 µl of cDNA, 10 µM forward and reverse primers, 5 µM probe, 7.5 µl of TaqMan master mix (Applied Biosystems, Foster City, CA, USA), with the following parameters: 95°C for 10 minutes, followed by 45 cycles of 95°C for 15 seconds and 60°C for 1 minute. The qPCR assays with a single pair of primers per reaction were done in a final volume of 10 µL with 3 µl of cDNA, 10 µM forward and reverse primers, 5 µM probe, 5 µl of TaqMan master mix, with the following parameters: 95°C for 10 minutes, followed by 45 cycles of 95°C for 15 seconds, and 60°C for 1 minute, except when the target was FLU A, HEV or HMPV A and B, when the parameters were: 95°C for 10 minutes, followed by 45 cycles of 95°C for 15 seconds, 55°C for 30 seconds, and 60°C for 1 minute. For the DNA viruses, qPCR was done with 300 ng of extracted DNA in a final volume of 10 µL, containing 10 µM forward and reverse primers, 5 µM probe and 5 µL of TaqMan master mix, using the same cycling parameters as for the RNA viruses. Applicable measures to prevent contamination of PCR reactions were taken in this study, including sample handling and reaction mix preparation in separate rooms. In addition, all qPCR plates included appropriate negative controls, matched to every step of clinical sample treatment. The negative controls were RT-qPCR products from RNA or DNA extracted from cultures of uninfected Hela cells; RT-qPCR product from ultrapure water; and just qPCR product from ultrapure water.

### Statistical Analysis

Chi-square and Exact Chi-square tests were used to compare patient groups and viral detection rates in relation to different parameters analyzed. Agreement between samples was tested using Kappa test, followed by the calculation of odds ratios. Analysis of presence of viruses in relation to ages and clinical conditions were done with Mann-Whitney test. Analyses were conducted using the SPSS software (Statistical Package for the Social Sciences), version 17(SPSS Inc.). A *P* value of ≤0.05 was chosen for significance.

## Results

### Virus Detection

**Figure 1:**
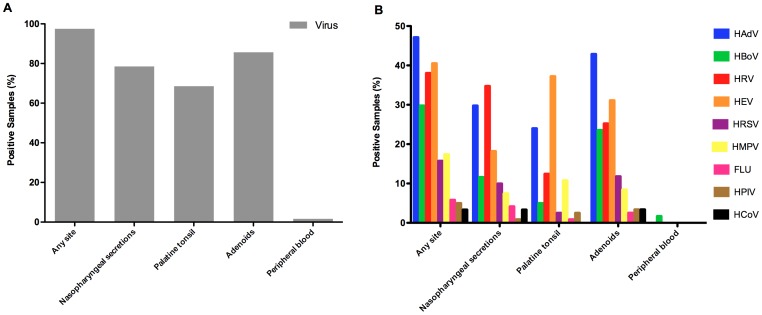
Frequencies of virus detection in patients with chronic adenotonsillar diseases. (**A**) Overall frequencies of virus detection by qPCR in different sample sites of 121 patients with chronic adenotonsillar disease. (**B**) Frequencies of detection of each tested virus by qPCR at different sites in 121 patients with chronic adenotonsillar disease.

This study included nasopharyngeal secretions (NS), fragments of tissue from adenoids and palatine tonsils and peripheral blood from a total of 121pediatric patients with indication of tonsillectomy due tonsillar hyperplasia or recurring tonsillitis. The overall frequency of virus detection was very high: 118 of 121 patients (97.5%) had at least one virus detected in at least one clinical sample (Figure 1a and Table 1). Only 3 patients had no respiratory virus detected by qPCR. The highest rate of virus detection was found in adenoids (85.7%), followed by NS (78.5%), palatine tonsils (68.6%), and peripheral blood (1%) (Figure 1a). Interestingly, when both kinds of tonsils were taken together, 112 (92.6%) of the patients were positive for at least one virus in at least one of the tonsils.

**Table 1:** Viral prevalence determined by qPCR in the different sample sites in 121 patients with chronic adenotonsillar disease.

VIRUS	Any Site*N (%)	Adenoids**N (%)	Palatine TonsilsN (%)	NSN (%)	Peripheral BloodN (%)
All Viruses#	118 (97.5)	102 (85.7)	83 (68.6)	95 (78.5)	2 (1.6)
HAdV	57 (47.1)	51 (42.8)	29 (23.9)	36 (29.8)	0 (0.0)
HBoV	36 (29.7)	28 (23.5)	6 (4.9)	14 (11.6)	2 (1.6)
HRV	46 (38.0)	30 (25.2)	15 (12.4)	42 (34.7)	0 (0.0)
HEV	49 (40.5)	37 (31.1)	45 (37.2)	22 (18.2)	0 (0.0)
HRSV	19 (15.7)	14 (11.8)	3 (2.5)	12 (9.9)	0 (0.0)
HMPV	21 (17.3)	10 (8.4)	13 (10.7)	9 (7.4)	0 (0.0)
FLU	7 (5.8)	3 (2.5)	1 (0.8)	5 (4.1)	0 (0.0)
HPIV	6 (4.9)	4 (3.7)	3 (2.5)	1 (0.8)	0 (0.0)
HCoV	4 (3.3)	4 (3.7)	0 (0.0)	4 (3.3)	0 (0.0)

* Any site: results of viral detection at any of the sites analyzed.

* Adenoids were obtained only from 119 of the total of 121 patients included in this study.

# All viruses: positivity for at least one virus by qPCR.

Considering the results of virus detection altogether, HAdV was the most often detected agent, in 57 (47.1%) patients, followed by HEV in 49 (40.5%), HRV in 46 (38%), HBoV in 36 (29.8%), HMPV in 21 (17.4%), HRSV in 19 (15.7%), FLU in 7 (5.8%), HPIV in 6 (4.9%) and HCoV in 4 (3.3%) (Figure 1b). Frequencies of detection of viruses in all sites are summarized in Table 1.

The detection of specific viruses notably varied among sample sites, indicating that respiratory viruses have different tissue tropisms, even when we compared pharyngeal and palatine tonsils. HAdV and HBoV were detected in adenoids more often than in others sites, whereas HEV and HRV were more frequently detected respectively in palatine tonsils and NS. Of the 119 patients from whom samples of all sites were obtained, 59 (49.6%) had at least one virus detected simultaneously at three sample kinds. In only 15 patients (12.6%) viruses were detected in only one sample (Table 2).

**Table 2:** Numbers of positive samples taken from different sites in 119 patients with chronic adenotonsillar disease from whom samples were obtained from all sites.

		PT +Ad +	PT +Ad −	PT −Ad +	PT −Ad −	Total*
**All viruses**	**NS+**	59	5	24	6	
	**NS−**	14	4	5	2	117
**HAdV**	**NS+**	18	1	17	0	
	**NS−**	5	5	11	62	57
**HBoV**	**NS+**	3	0	5	6	
	**NS−**	1	2	19	83	36
**HRV**	**NS+**	14	0	12	16	
	**NS−**	1	0	3	73	46
**HEV**	**NS+**	20	1	1	0	
	**NS−**	13	11	3	70	49

Adenoids were not available from two patients, one of whom was negative for all tested viruses, while the other had HRSV in NS.

PT: palatine tonsil; Ad: adenoid; NS: nasopharyngeal secretion.

* Total number of positive samples.

The virus most frequently detected in NS was HRV, in 42 (34.7%) patients, followed by HAdV (29.8%), HEV (18.2%), HBoV (11.6%), and all other viruses were detected in frequencies lower than 10%. In the palatine tonsils, HEV was found in 45 samples (37.2%), followed by HAdV in 29 samples (24%), HRV in 15 samples (12.4%), and all other viruses were detected in frequencies lower than 10%.

Overall virus detection rates were notably higher in adenoids, where the most frequent virus detected was HAdV in 42.9%, followed by HEV in 37 (30.6%), HRV in 30 (24.8%) and HBoV in 28 (23.1%) patients. The only virus detected by qPCR in the peripheral blood was HBoV, found in two patients (Figure 1b).

HAdV was more frequently detected in the adenoids, although simultaneous detection in other sampling sites was very frequent. Only 6 of all 57 HAdV-positive patients had the virus detected only in sites different from the adenoids (Table 2). The kappa value for agreement between HAdV detection in adenoids and palatine tonsils was 0.4 (95% confidence interval: 0.24–0.56, p<0.001), and for detection in adenoids and secretions it was 0.71 (95% confidence interval: 0.58 to 0.84, p<0.001), respectively. Detection of HAdV in adenoid increased by more than 10 times the chance of detecting it in palatine tonsils (OD: 10.35, 95% confidence interval: 3.57 to 30.01, p<0.001) and NS (OD: 294.76; 95% confidence interval:17.17 to 5061.00, p<0.001).

Similarly, HBoV was also more frequently detected in adenoids than in other sites, and 52.8% of patients had HBoV detectable only in the adenoid tissue. Detection of HBoV in adenoids was associated with simultaneous detection of that virus in palatine tonsils (kappa = 0.17; 95% confidence interval: 0.01–0.35; p = 0.03; OD = 7.42; 95% confidence interval: 1.28–42.95; p = 0.01) and NS (kappa = 0.27; 95% confidence interval: 0.06–0.47; p = 0.004; OD = 5.67; 95% confidence interval: 1.78–18.17; p = 0.002). In just 2 patients HBoV was detected only in the palatine tonsils, but not in the adenoids.

NS was the only sample positive for HRV in 34.8% of patients, and in only 4 patients HRV was detected exclusively in samples other than NS (Table 2). Detection HRV in NS was associated with its presence in both palatine tonsils [kappa = 0.38, 95% confidence interval: 0.22 to 0.54; p<0.001) and adenoids [kappa = 0.61; 95% confidence interval: 0.46 to 0.76; p<0.001). Detection of HRV in nasopharyngeal secretions increased the chance of detecting this virus by more than 29 times for adenoids (relative risk = 29.66; 95% confidence interval: 9.08 to 96.86; p<0.001) and 39 times for tonsils (relative risk = 39.00; 95% confidence interval: 4.90 to 310.37; p<0.001).

While HAdV and HBoV were more frequently detected in adenoids, enteroviruses were significantly more frequent in palatine tonsils. Of 49 patients positive for HEV, 45 (92%) had positivity in palatine tonsils, which was strongly correlated with their presence in adenoids (kappa = 0.7; confidence interval 0.54 to 0.84; p<0.001) and nasopharyngeal secretions (kappa = 0.51; confidence interval: 0.35 to 0.67; p<0.001). Thus, the detection of HEV in palatine tonsils increased the chance of finding HEV in adenoids by 48.13 times (95% confidence interval: 14.42 to 160.57; p<0.001) and in NS by 65.63 times (95% confidence interval: 8.38 to 513.90; p<0.001). Interestingly, in 11 of 45 (24.5%) patients with HEV detectable in palatine tonsils, that site was the only one positive for HEV, as opposed to 4 (8.8%) patients in whom HEV was detected solely in sites other than palatine tonsils (Table 2).

For all remaining viruses the rates of detection were lower, hampering comparisons between sampling sites.

### Viral-coinfections

**Figure 2:**
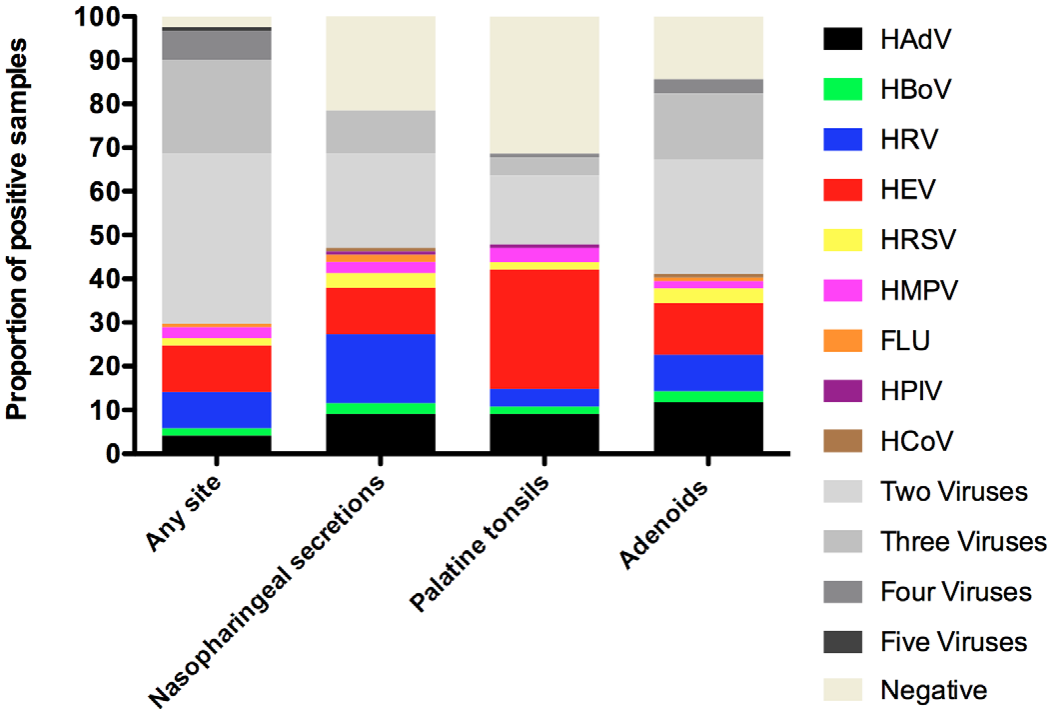
Frequencies of virus co-infections in different sample sites in 121 patients with chronic adenotonsillar diseases.

High rates of viral co-infections were obtained in this patient group. Of 118 patients positive for any respiratory virus, 82 (69.5%) had more than one virus. Of the 82 patients who had more than one detectable agent, 47 (57%) had 2, 26 (32%) had 3, 8 (9.7%) had 4, and 1 patient had five simultaneously detectable viruses (Figure 2). The viruses most frequently detected as single agents were HEV in 13 (11%), followed by HRV in 10 (8.5%) and HAdV in 5 (4.3%) patients, respectively. Other viruses were found as single agents in frequencies lower than 3% (Table 3).

**Table 3:** Frequencies of specific viruses and viral associations at different sample sites in 121 patients with chronic adenotonsillar diseases.

Virus Detection		Any Site* N (%)	NS N (%)	PT N (%)	Ad** N (%)
**Single infection**	HAdV	5 (4.1)	11 (9.1)	11 (9.1)	14 (11.8)
	HBoV	2 (1.6)	3 (2.5)	2 (1.6)	3 (2.5)
	HRV	10 (8.2)	19 (15.7)	5 (4.1)	10 (8.4)
	HEV	13 (10.7)	13 (10.7)	33 (27.3)	14 (11.8)
	HRSV	2 (1.6)	4 (3.3)	2 (1.6)	4 (3.3)
	HMPV	3 (2.5)	3 (2.5)	4 (3.3)	2 (1.6)
	FLU	1 (0.8)	1 (0.8)	0 (0.0)	1 (0.8)
	HPIV	0 (0.0)	2 (1.6)	1 (0.8)	0 (0.0)
	HCoV	0 (0.0)	1 (0.8)	0 (0.0)	1 (0.8)
	**TOTAL**	**36 (29.7)**	**57 (47.1)**	**58 (47.9)**	**49 (41.1)**
**Dual infection**	HAdV + HRV	8 (6.6)	9 (7.4)	4 (3.3)	6 (5.0)
	HAdV + HEV	9 (7.4)	1 (0.8)	7 (5.8)	8 (6.6)
	HBoV + HEV	7 (5.8)	2 (1.6)	0 (0.0)	3 (2.5)
	HEV + HRSV	4 (3.3)	0 (0.0)	0 (0.0)	4 (3.3)
	HBoV + HRV	3 (2.5)	1 (0.8)	1 (0.8)	1 (0.8)
	HAdV + HBoV	2 (1.6)	3 (2.5)	1 (0.8)	3 (2.5)
	HAdV + HRSV	3 (2.5)	2 (1.6)	0 (0.0)	1 (0.8)
	HRV + HMPV	2 (1.6)	2 (1.6)	2 (1.6)	0 (0.0)
	HRV + HCoV	2 (1.6)	2 (1.6)	0 (0.0)	1 (0.8)
	HAdV + HMPV	1 (0.8)	0 (0.0)	1 (0.8)	1 (0.8)
	HBoV + HRSV	1 (0.8)	0 (0.0)	0 (0.0)	1 (0.8)
	HBoV + FLU	1 (0.8)	0 (0.0)	0 (0.0)	1 (0.8)
	HRV + HPIV	1 (0.8)	0 (0.0)	0 (0.0)	0 (0.0)
	HRV + FLU	1 (0.8)	1 (0.8)	0 (0.0)	0 (0.0)
	HEV + HMPV	1 (0.8)	1 (0.8)	1 (0.8)	0 (0.0)
	HEV + HPIV	1 (0.8)	0 (0.0)	0 (0.0)	1 (0.8)
	HMPV + HPIV	0 (0.0)	0 (0.0)	1 (0.8)	0 (0.0)
	HRV + HRSV	0 (0.0)	1 (0.8)	1 (0.8)	0 (0.0)
	HRSV + FLU	0 (0.0)	1 (0.8)	0 (0.0)	0 (0.0)
	**TOTAL**	**47 (38.8)**	**26 (21.5)**	**19 (15.7)**	**31 (26.0)**
**Triple Infection**	HAdV + HBoV + HEV	4 (3.3)	2 (1.6)	1 (0.8)	3 (2.5)
	HAdV + HBoV + HRV	5 (4.1)	1 (0.8)	0 (0.0)	5 (4.2)
	HAdV + HRV + HMPV	6 (4.9)	2 (1.6)	2 (1.6)	3 (2.5)
	HAdV + HEV + HMPV	2 (1.6)	1 (0.8)	2 (1.6)	0 (0.0)
	HAdV + HRV + HRSV	2 (1.6)	2 (1.6)	0 (0.0)	2 (1.6)
	HAdV + HBoV + HRSV	1 (0.8)	1 (0.8)	0 (0.0)	0 (0.0)
	HAdV + HEV + FLU	1 (0.8)	1 (0.8)	0 (0.0)	0 (0.0)
	HAdV + HEV + HPIV	1 (0.8)	0 (0.0)	0 (0.0)	0 (0.0)
	HAdV + HRSV + FLU	1 (0.8)	0 (0.0)	0 (0.0)	0 (0.0)
	HBoV + HRV + HRSV	1 (0.8)	0 (0.0)	0 (0.0)	0 (0.0)
	HBoV + HMPV + HPIV	1 (0.8)	0 (0.0)	0 (0.0)	1 (0.8)
	HEV + HRSV + FLU	1 (0.8)	0 (0.0)	0 (0.0)	0 (0.0)
	HBoV + HRV + HCoV	0 (0.0)	1 (0.8)	0 (0.0)	1 (0.8)
	HBoV + HPIV + FLU	0 (0.0)	0 (0.0)	0 (0.0)	1 (0.8)
	HAdV + HBoV + HMPV	0 (0.0)	0 (0.0)	0 (0.0)	1 (0.8)
	HBoV + HEV + HPIV	0 (0.0)	0 (0.0)	0 (0.0)	1 (0.8)
	HRV + HEV + HRSV	0 (0.0)	1 (0.8)	0 (0.0)	0 (0.0)
	**TOTAL**	**26 (21.5)**	**12 (9.9)**	**5 (4.1)**	**18 (14.8)**
**Quadruple infection**	HAdV + HBoV + HRV + HMPV	2 (1.6)	0 (0.0)	0 (0.0)	0 (0.0)
	HAdV + HBoV + HEV + HCoV	1 (0.8)	0 (0.0)	0 (0.0)	1 (0.8)
	HAdV + HBoV + HEV + HMPV	1 (0.8)	0 (0.0)	0 (0.0)	1 (0.8)
	HAdV + HBoV + HRV + HRSV	1 (0.8)	0 (0.0)	0 (0.0)	1 (0.8)
	HAdV + HEV + HRSV + HMPV	1 (0.8)	0 (0.0)	0 (0.0)	1 (0.8)
	HBoV + HRV + HMPV + HCoV	1 (0.8)	0 (0.0)	0 (0.0)	0 (0.0)
	HBoV + HEV + HPIV + FLU	1 (0.8)	0 (0.0)	1 (0.8)	0 (0.0)
	**TOTAL**	**8 (6.6)**	**0 (0.0)**	**1 (0.8)**	**4 (3.3)**

One patient had co-infection with five viruses: HBoV + HRV + HEV + HRSV + HPIV; PT: palatine tonsil; Ad: adenoid tissue; NS: nasopharyngeal secretion.

* The indicated single virus or combination was the only identified at any of the sites in the patient.

** Adenoids were obtained only from 119 of the total of 121 patients included in this study.

The rates of detection of more than one virus per sample site were lower in palatine tonsils than in adenoids (44.5%) and NS (31.4%) (Figure 2). In only 25 patients (20.7%), viral co-infections were detected in palatine tonsils. The higher frequency of HEV as exclusive agent was in palatine tonsils, where HEV was detected alone in 33 patients (27.3%) (Figure 2). The viruses most frequently detected as single agents in NS and adenoids were respectively: HRV in 19 (15.9%) patients, and HAdV in 14 (11.6%) patients.

**Figure 3:**
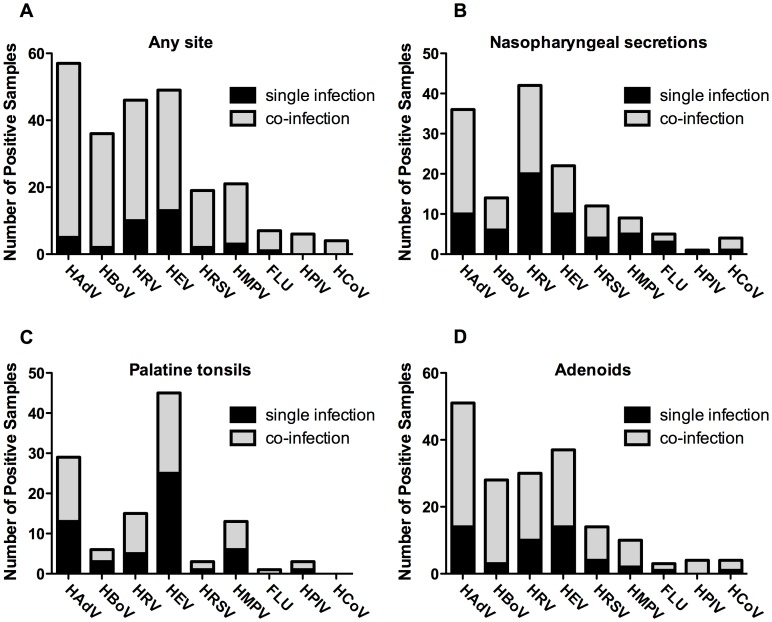
Frequencies of virus co-infections associated with each analyzed viruses at different sample sites in 121 patients with chronic adenotonsillar diseases. (**A**) Overall number of virus co-infections. (**B**) Number of virus co-infections detected in nasopharyngeal secretions. (**C**) Number of virus co-infections detected in palatine tonsils. (**D**) Number of virus co-infections detected in adenoids.

Although the frequencies of viral co-infections for specific viruses varied among sample sites, DNA viruses (HAdV and HBoV) were more often detected as part of co-infections than RNA viruses (Figure 3). HBoV was the single most frequently detected in co-infection with other agents regardless of sample site (Table 3). Conversely, HEV was the least frequently detected virus in co-infection with other agents, in particular in palatine tonsils (Figure 3).

The virus co-infections observed in this study generally involved DNA viruses in combination with some RNA virus, most notably HRV and HEV (Table 3). The most frequent co-infection detected was HAdV with HEV, in 9 patients, of whom 8 had HAdV in the adenoid and HEV in the palatine tonsil (Table 3). Other frequent co-infections were of HRV (usually in the NS) with HAdV (usually in adenoid); and of HBoV (usually in adenoids) with HEV (usually in palatine tonsils). It is noteworthy that of 82 patients in whose samples more than one virus were detected, only 13 (15.8%) had co-infections involving exclusively RNA viruses.

Frequencies of specific combinations of co-infecting viruses varied among sample sites. In adenoids, co-infections more frequently involved HAdV + HEV, HAdV + HRV, and HAdV + HBoV + HRV, respectively in 8 (6.7%), 6 (5%) and 5 (4.2%) patients. The most frequent virus associations in palatine tonsils and NS were, respectively, HAdV + HEV, and HAdV + HRV.

### Clinical Features

All patients enrolled in this study underwent tonsillectomy due to adenotonsillar hypertrophy or recurring tonsillitis. Of 121 patients, 16 (12.4%) also had otitis media with effusion, 61 (50.5%) had apnea, and 33 (27.3%) reported underlying conditions, including asthma in 5 and symptoms of allergy in 19.

The presence of allergic symptoms, bronchial asthma and recurrent otitis media with effusion were not significantly associated with the presence of specific respiratory viruses, except for an association of HRSV in adenoids with reported allergy (χ^2^ = 4.61, p = 0.04).

Although global virus detection rates in adenoids and palatine tonsils were higher in younger patients (Z = 2.34, p = 0.02 for adenoids, and Z = 2.79, p = 0.005 for palatine tonsils), when that analysis was done for specific agents, significance was noted only for HBoV (Z = 3.67, p<0.005).

Virus detection, both overall and agent-specific, was not associated with recurrent tonsillitis (Table 4). In fact, rates of virus detection were not significantly different (χ^2^ = 3.01, p = 0.10) in children with recurrent tonsillitis and/or severe tonsillar hypertrophy (>70%), as compared to children without recurrent tonsillitis and in those with lower degrees of palatine tonsil hypertrophy (45.45%).

**Table 4:** Demographic data and virus detection frequencies in patients according to categories of adenotonsillar disease.

Clinical feature/virus detection	RTN (%)	THN (%)	Non RT-THN (%)	RT/THN (%)
Patients (N)	11 (9.09)	49 (40.49)	11 (9.09)	50 (41.33)
Females	7 (63.63)	22 (44.89)	2 (18.18%)	27 (54%)
Age (mean in years)	5.91	5.85	7.09	6.33
All Viruses +	11 (100)	48 (97.96)	10 (90.90)	49 (98)
virus co-infections +	8 (72.72)	33 (67.34)	7 (63.63)	34 (68)
Virus + in palatine tonsils	8 (72.72)	34 (69.38)	5 (45.45)	36 (72)
Virus co-infections in palatine tonsils	1 (9.09)	8 (16.32)	4 (36.36)	12 (24)
HAdV +	5 (45.45)	20 (40.81)	6 (54.54)	26 (52)
HAdV + isolated	1 (9.09)	1 (2.04)	1 (9.09)	2 (4)
HAdV + in palatine tonsils	3 (27.27)	10 (20.41)	3 (27.27)	13 (26)
HAdV + isolated in palatine tonsils	2 (18.18)	2 (4.08)	0 (0)	7 (14)
HBoV +	3 (27.27)	16 (32.65)	1 (9.09)	16 (32)
HBoV + isolated	0 (0)	1 (2.05)	0 (0)	1 (2)
HBoV + in palatine tonsils	1 (9.09)	2 (4.08)	0 (0)	3 (6)
HBoV + isolated in palatine tonsils	1 (9.09)	0 (0)	0 (0)	1 (2)
HRV +	3 (27.27)	16 (32.65)	4 (36.36)	23 (46)
HRV + isolated	2 (18.18)	5 (10.20)	0 (0)	3 (6)
HRV + in palatine tonsils	1 (9.09)	4 (8.16)	3 (27.27)	7 (14)
HRV + isolated in palatine tonsils	1 (9.09)	3 (6.12)	0 (0)	1 (2)
HEV +	5 (45.45)	23 (46.94)	2 (27.27)	19 (38)
HEV + isolated	0 (0)	6 (12.24)	0 (0)	7 (14)
**HEV + in palatine tonsils** *	**4 (36.36)**	**23 (46.94)**	**1 (9.09)**	**17 (34)**
**HEV + isolated in palatine tonsils** *	**3 (27.27)**	**18 (36.73)**	**0 (0)**	**12 (24)**
HRSV +	3 (27.27)	8 (16.32)	3 (27.27)	5 (10)
HRSV + isolated	0 (0)	1 (2.04)	0 (0)	1 (2)
HRSV + in palatine tonsils	0 (0)	1 (2.04)	1 (9.09)	1 (2)
HRSV + isolated in palatine tonsils	0 (0)	1 (2.04)	0 (0)	1 (2)
HMPV +	1 (9.09)	3 (6.12)	3 (27.27)	14 (28)
HMPV + isolated	0 (0)	1 (2.04)	1 (9.09)	1 (2)
HMPV + in palatine tonsils	0 (0)	2 (4.08)	2 (18.18)	9 (18)
HMPV + isolated in palatine tonsils	0 (0)	1 (2.04)	1 (9.09)	2 (4)
FLU +	1 (9.09)	4 (8.16)	1 (9.09)	1 (2)
FLU + isolated	0 (0)	0 (0)	1 (9.09)	0(0)
FLU + in palatine tonsils	0 (0)	0 (0)	0 (0)	1(2)
FLU + isolated in palatine tonsils	0 (0)	0 (0)	0 (0)	0(0)
HPIV +	0 (0)	3 (6.12)	0 (0)	3 (6)
HPIV + isolated	0 (0)	0 (0)	0 (0)	0 (0)
HPIV + in palatine tonsils	0 (0)	1 (2.04)	0 (0)	2 (4)
HPIV + isolated in palatine tonsils	0 (0)	1 (2.04)	0 (0)	0 (0)
HCoV +	0 (0)	1 (2.04)	0 (0)	3 (6)
HCoV + isolated	0 (0)	0 (0)	0 (0)	0 (0)
HCoV + in palatine tonsils	0 (0)	0 (0)	0 (0)	0 (0)
HCoV + isolated in palatine tonsils	0 (0)	0 (0)	0 (0)	0 (0)

RT: Recurrent Tonsillitis; TH: Tonsilar Hypertrophy (Brodsky grade >3); non RT/TH: patients with smaller size tonsils (less than Brodsky 3) and without recurrent tonsillitis; RT/TH: Recurrent Tonsillitis and Tonsilar Hypertrophy.

* p≤0.05.

Overall rates of virus detection were not significantly different between patients with large (Brodsky ≥3) and small palatine tonsils (Brodsky ≤3) (χ^2^ = 3.58, p = 0.06). However, there was an association between higher virus detection rates with larger palatine tonsils in the specific case of HEV, since this agent was detected in palatine tonsils from 40 of 98 (40.8%) patients with higher hypertrophy degrees (Brodsky ≥3), and in 5 of 22 (22.7%) patients with less severe hypertrophy (Brodsky <3) (χ^2^ = 4.09, p = 0.05). Rates of detection of other viruses were similar between patients with larger and smaller palatine tonsils (Table 4).

Overall rates of virus detection in adenoids were higher in patients with more severely enlarged adenoids (Table 5). The proportion of patients with larger adenoids (nasopharyngeal obstruction ≥75%) was significantly higher (χ^2^ = 3.78, p = 0.05) in patients with virus detected in the adenoidal tissue (43/102, 42.2%) than in patients without virus in adenoids (3/17, 17.6%). Patients with virus in adenoid tissue had a chance 3.4 times higher of having a high degree of adenoid hypertrophy (75–100%) than those without virus detected there (OD = 3.4; confidence interval: 0.92–12.57; p = 0.05). Although HEV was frequently detected in adenoids from patients with nasopharyngeal obstruction ≥75% (χ^2^ = 3.65, p = 0.06), no single virus was individually associated with large adenoids (75% to 100% nasopharyngeal obstruction).

**Table 5:** Demographic data and virus detection frequencies in patients according to adenoid sizes.

Clinical feature/virus detection	0–50%	50–75%	75–100%
Patients (N)	15 (10.08)	58 (48.74)	46 (38.66)
Females	4 (26.66)	31 (55.5)	22 (47.82)
**Age (mean in years)** *	**7.0**	**6.31**	**5.26**
All Viruses +	15(100)	56 (96.55)	46 (100)
virus co-infections +	11 (73.33)	35 (60.34)	35 (76.08)
**Virus + in adenoids** *	**11 (73.3)**	**48 (82.75)**	**43 (93.47)**
Virus co-infections in adenoids	5 (33.33)	21 (36.20)	27 (58.69)
HAdV +	7 (46.66)	28 (48.27)	21(45.65)
HAdV + isolated	1 (6.66)	2 (3.44)	2 (4.34)
HAdV + in adenoids	7 (46.66)	24 (41.38)	20 (43.47)
HAdV + isolated in adenoids	4 (26.66)	7 (12.06)	3 (6.52)
HBoV +	4(26.66)	17 (29.31)	15 (32.60)
HBoV + isolated	0 (0)	2 (3.44)	0 (0)
HBoV + in adenoids	3 (20.0)	13 (22.41)	12 (26.08)
HBoV + isolated in adenoids	0 (0)	2 (3.44)	1 (2.17)
HRV +	8 (53.33)	22 (37.93)	16 (34.78)
HRV + isolated	3 (20.0)	5 (8.62)	2 (4.34)
HRV + in adenoids	3 (20.0)	17 (29.31)	10 (21.74)
HRV + isolated in adenoids	1 (6.66)	7 (12.06)	2 (4.34)
HEV +	7 (46.66)	21 (36.20)	21 (45.65)
HEV + isolated	0 (0)	8 (13.79)	5 (10.87)
**HEV + in adenoids**	**3 (20.0)**	**15 (25.86)**	**19 (41.30)**
HEV + isolated in adenoids	0 (0)	9 (15.51)	5 (10.87)
HRSV +	2 (13.33)	7 (12.06)	9 (19.56)
HRSV + isolated	0 (0)	1 (1.72)	1 (2.17)
HRSV + in adenoids	0 (0)	6 (10.34)	8 (17.39)
HRSV + isolated in adenoids	0 (0)	1 (1.72)	3 (6.52)
HMPV +	4 (26.66)	7 (12.06)	10 (21.74)
HMPV + isolated	0 (0)	2 (3.44)	1 (2.17)
HMPV + in adenoids	2 (13.33)	1 (1.72)	7 (15.21)
HMPV + isolated in adenoids	1 (6.66)	0 (0)	1 (2.17)
FLU +	1 (6.66)	5 (8.62)	1 (2.17)
FLU + isolated	0 (0)	1 (1.72)	0 (0)
FLU + in adenoids	0 (0)	3 (5.17)	0 (0)
FLU + isolated in adenoids	0 (0)	1 (1.72)	0 (0)
HPIV +	2 (13.33)	1 (1.72)	3 (6.52)
HPIV + isolated	0 (0)	0 (0)	0 (0)
HPIV + in adenoids	1 (6.66)	1 (1.72)	2 (4.34)
HPIV + isolated in adenoids	0 (0)	0 (0)	0 (0)
HCoV +	0 (0)	1 (1.72)	3 (6.52)
HCoV + isolated	0 (0)	0 (0)	0 (0)
HCoV + in adenoids	0 (0)	1 (1.72)	3 (6.52)
HCoV + isolated in adenoids	0 (0)	0 (0)	1 (2.17)

* p≤0.05.

Obs: Adenoids were not available from two patients.

## Discussion

To the best of our knowledge, this has been the most comprehensive study of respiratory viruses in chronic adenotonsillar diseases of children so far conducted. The study was based on sensitive molecular assays to detect respiratory viruses in sets of samples prospectively collected from children with a full spectrum of chronic adenotonsillar diseases requiring tonsillectomy. The observed frequency of respiratory virus genomes detected in 97.5% of the children is remarkably high. Such staggering observation is novel and should be regarded in the light of the high prevalence of adenotonsillar diseases, pondering the possible roles that persistence of common respiratory virus may play on their pathogenesis.

Testing the nucleic acid extracts obtained from macerated fragments of upper respiratory lymphoepithelial tissues by qPCR revealed an array of respiratory viral genomes in over 85% of adenoids and close to 70% of palatine tonsils. Considering that the children had no symptoms of acute respiratory infections, these proportions of detection in tissues are by all means extremely high.

Lymphoid tissues associated with the upper respiratory tract have been recognized as sites of latency/persistency of such agents as Epstein-Barr virus, human herpes virus 6, HIV, measles virus, picornaviruses, human bocavirus and human papillomavirus-16 [4], [5], [10]–[14]. However, large studies of the presence of the most common respiratory viruses in lymphoepithelial tissues damaged by chronic inflammation or hypertrophy are virtually lacking, what underscores the novelty of the presently reported findings.

While detection of DNA virus genomes (HAdV and HBoV) in association with lymphoepithelial organs is not novel, such is not the case of detecting respiratory RNA viruses, for which the existence of persistency and latency is far from clear. Therefore, the most surprising findings were the high frequencies of detection of the picornaviruses HRV and HEV, both detected in approximately 40% of the children, in the absence of discernible clinical features of acute respiratory infection. When HRV and HEV were taken together, the frequencies of picornavirus detection in adenoids and palatine tonsils were respectively 55.4% and 49.6%, showing that these agents together can be present in lymphoepithelial tissues in up to one half of subjects with chronic tonsillar hypertrophy. However, when considering HRV and HEV separately, as made possible with specific primers and probes by qPCR, interesting distinct features become evident.

Detection of HRV in nasopharyngeal secretions was highly frequent (34.7%), and was associated with an increase of 30–40 times in the chance of finding it also in tonsils. Indeed, in only 4 of 46 HRV-positive patients (8.7%), the virus was detected only in tonsils, without concurrent detection in nasopharyngeal secretions. In contrast, detection of HEV in palatine tonsils was significantly more frequent than in adenoids and nasopharyngeal secretions and, conversely, in only 4 of 49 HEV-positive patients (8.2%) the virus was detected in sampling sites either than palatine tonsils. A tropism of HEV for palatine tonsils is illustrated by this site being the only HEV-positive site in 25% of all 45 patients with HEV in palatine tonsils.

The frequent finding of picornaviruses in adenoid and palatine tonsil tissues in the present study expands on findings of a previous study done in Finland [10]. In that study a very high frequency of detection of picornavirus RNA (75%) was found by in situ hybridization, yet in a restricted number of palatine tonsil tissues from patients with chronic tonsillar disease. Of note, that study pointed towards a role for HRV in pathogenesis of chronic tonsillitis based on the analysis of tissues from 4 patients. Moreover, the in situ hybridization signal attributed to HRV, could have been caused by persistent HEV. The specificity of our findings is supported by the TaqMan technology, which relies on the hybridization of a fluorescent probe internal to the amplicon, bringing reliability to the differentiation between HRV and HEV. Detection of picornaviruses in that study appeared to be more frequent in patients with chronic tonsillitis than in patients with recurrent tonsillitis, what is in agreement with our observations.

Not surprisingly, HAdV was the most frequently detected agent, present in near one half of all patients when results from all sample sites were taken together; and in over 42% considering only macerated adenoid tissues. In fact, HAdV was first identified in association with adenoid tissue [8], and our findings confirm the strong tropism of that agent for lymphoepithelial tissues. Although the molecular details of HAdV persistency are not completely understood [15], the prolonged presence of this agent in nasopharyngeal tonsils allows for periodic and asymptomatic virus shedding that ensures successful transmission with infection of vast proportions of humans at some point in life [16], [17].

HBoV is a recently identified parvovirus, found in association with respiratory and gastrointestinal disorders [18], [19]. We have shown previously that only approximately 25% of patients with detectable HBoV genome shed mRNA, a marker of active viral replication [20], indicating that a significant proportion of infected HBoV-patients carry the virus in a latent/persistent state. This is indirectly supported by the fact that bocaviruses of other species can persist in lymphoid tissues of the upper respiratory tract, indicating that HBoV could persist in humans in a similar way and be related to chronic inflammation of these tissues. In fact, a recent flow cytometry study has shown that HBoV can be detected in tonsillar lymphocytes in 32% of pediatric patients who underwent tonsillectomy [13]. In agreement with that, the presently reported study found HBoV in nearly 24% of the adenoids, and further expanded to reveal that HBoV is more likely to be detected in in adenoids than in palatine tonsils or nasopharyngeal secretions, suggesting a preference for adenoids over palatine tonsils and thus bringing new insights to the pathogenesis of this emergent virus.

This is the first report of viral co-infection in chronic adenotonsillar diseases, and it is notable that two thirds of the patients had more than one respiratory virus detected simultaneously, even in the absence of symptoms and signs of acute respiratory infection. The majority of viral co-infections resulted from combinations of HAdV, HBoV, HEV and HRV, and co-infections were more frequent when detections of viruses both in tissues and nasopharyngeal secretions were considered. When only adenoids and palatine tonsils were analyzed, rates of viral coinfections were close to 45% and 21%, respectively.

The presently reported findings on co-infections are similar to what has been known for acute respiratory infections, which can yield as much as 40% to 70% of virus co-detection [21], [22]. Martin et al have shown that viral co-infections rates are higher in patients with HBoV and HAdV than in those with HRV and other RNA viruses. They reported rates of co-infections of 72% in patients with HBoV, and of 33% in patients with HRV.

No clear associations were found between any particular virus and clinical features. However, it should be pointed out that there was a high frequency of virus detection, especially HEV, in highly hypertrophic tissues. This is in keeping with results published for EBV [4], showing a significant difference in frequency of positive results for EBV by *in situ* hybridization in younger patients with higher degrees of palatine tonsil hypertrophy than in older patients with a lower degrees of hypertrophy.

While mechanisms that might underlie a link of the presence of prolonged viral infection in tonsils with chronic tonsillar diseases, it is tempting to speculate that virus products may function as pathogen-associated molecular patterns (PAMPs), that would maintain inflammation by the stimulation of the innate immunity, with continuous processes of damage and repair, thus leading to hyperplasia and hypertrophy of tonsils, whose enlarged sizes would cause clinical features of chronic tonsillar disease. This finds support on histological studies of enlarged tonsils, which reveals enlargement of follicles, indicating hyperplasia with marked increase in the numbers of lymphoid cells in the germinal centers [23]. On the other hand, it is possible that some respiratory viruses may directly affect tonsil cell survival, by counteracting natural mechanisms of cell death, including apoptosis. Reduction in lymphoid cell apoptosis is accepted as an important mechanism in the pathogenesis of tonsillar hypertrophy [24] **,** and that could be caused by viruses, such as the case of human coronavirus, which has a nonstructural protein that inhibits apoptosis triggered by the mitochondrial antiviral signaling adaptor (MAVS) [25].

In summary, the present study has shown that a very high proportion of patients with chronic adenotonsillar diseases harbor at least one respiratory virus, and that these agents are frequently detected in adenotonsillar tissues. The patients were sampled only once, which hampers determination of the duration of virus presence in them. Nevertheless, significant proportions of such patients had different combinations of viruses simultaneously detected at different sites, suggesting that some of them might be persistent. It is remarkable that not all respiratory viruses were detected at similar frequencies in adenoids and palatine tonsils, nor were uniformly shed in nasopharyngeal secretions. Specific viruses seem to preferentially infect some tissues, such as human enteroviruses that were more often detected in palatine tonsils than in adenoids, and were shed in secretions much less frequently than all others. Taken together, these data suggest that respiratory virus persistence/latency may play roles in pathogenesis of chronic adenotonsillar diseases, and that these tissues may serve as inter-seasonal virus reservoirs, with possible impact in transmission in the community.

## Supporting Information

Table S1 Primers and probes used for qPCR.
(DOCX)
