# Supplementary material for: High Rates of Detection of Respiratory Viruses in Tonsillar Tissues from Children with Chronic Adenotonsillar Disease
Source: PLoS One. 2012 Aug 3;7(8):e42136. doi: 10.1371/journal.pone.0042136 (PMC3411673; doi:10.1371/journal.pone.0042136)
Supplement: Table S1 — Primers and probes used for qPCR. (DOCX) [file pone.0042136.s001.docx]

**Table S1.** Primers and probes used for qPCR.

| **Virus or housekeeping gene** | **Primer** | **Sequence** | **Target** | **Reference** |
| --- | --- | --- | --- | --- |
| **HRSV** | A21-F | GCTCTTAGCAAAGTCAAGTTGAATGA | N | [1] |
|  | A102-R | TGCTCCGTTGGATGGTGTATT | N |  |
|  | APB48 | Fam-ACACTCAACAAAGATCAACTTCTGTCATCCAGC-Tamra | N |  |
|  | B17-F | GATGGCTCTTAGCAAAGTCAAGTTAA | N |  |
|  | B120-R | TGTCAATATTATCTCCTGTACTACGTTGAA | N |  |
|  | BPB45 | Joe-TGATACATTAAATAAGGATCAGCTGCTGTCATCCA-Tamra | N |  |
|  |  |  |  |  |
| **HMPV** | HMPV-F | GTGATGCACTCAAGAGATACCC | N | [2] |
|  | HMPV-R | CATTGTTTGACCGGCCCCATAA | N |  |
|  | HMPV-probe | Fam-CTTTGCCATACTTCAATGAACAAC-Tamra | N |  |
|  |  |  |  |  |
| **HEV** | HEV-F | GCGGAACCGACTACTTTGGG | 5´UTR | Developed in the present study. |
|  | HEV-R | CTCAATTGTCACCATAAGCAGCC | 5´UTR |  |
|  | HEV-probe | Fam-TCCGTGTTTCCTTTTATTCTTATA-MGB | 5´UTR |  |
|  |  |  |  |  |
| **HRV** | HRV-F | GCACTTCTGTTTCCCC | 5´UTR | [3] |
|  | HRV-R | GGCAGCCACGCAGGCT | 5´UTR |  |
|  | HRV-probe 1 | Fam-AGCCTCATCTGCCAGGTCTA-Tamra | 5´UTR |  |
|  | HRV-probe 2 | Vic-AGCCTCATCGACCAAACT- Tamra | 5´UTR |  |
|  |  |  |  |  |
| **HCoV** | HCoV-F3 | TGGCGGGTGGGATAATATGT | Pol | [4] |
|  | HCoV-ocF | CCTTATTAAAGATGTTGACAATCCTGTAC | Pol |  |
|  | HCoV-R3 | GAGGGCATAGCTCTATCACACTTAGG | Pol |  |
|  | HCoV-ocR | AATACGTAGTAGGTTTGGCATAGCAC | Pol |  |
|  | HCoV-P2 | Fam-ATAGTCCCATCCCATCAA-Tamra | Pol |  |
|  | HCoV-Poc | Fam-CACACTTAGGATAGTCCCA-Tamra | Pol |  |
|  |  |  |  |  |
| **HPIV** | Para1F | CATTATCAATTGGTGATGG | HN | [5] |
|  | Para1R | CTTAAATTCAGATATGTATCCTG | HN |  |
|  | Para1-probe | Fam-CTTAATCACTCAAGGATGTGCAGATATA-Tamra | HN |  |
|  | Para3F | CTCGAGGTTGTCAGGATATAG | HN |  |
|  | Para3R | CTTGAGGTTGTCAGGATATT | HN |  |
|  | Para3-probe | Fam-AATAACTGTAAACTCAGACTTGGTACCTGACTT-Tamra | HN |  |
|  |  |  |  |  |
| **FLU** | InfA for | GACCRATCCTGTCACCTCTGAC | M | [6] |
|  | InfArev | AGGGCATTYTGGACAAAKCGTCTA | M |  |
|  | InfAprobe | Fam-TGCAGTCCTCGCTCACTGGGCACG-BHQ1 | M |  |
|  | INFB-1 | AAATACGGTGGATTAAATAAAAGCAA | HA | [7] |
|  | INFB-2 | CCAGCAATAGCTCCGAAGAAA | HA |  |
|  | INFB-probe | Vic-CACCCATATTGGGCAATTTCCTATGGC-Tamra | HA |  |
|  |  |  |  |  |
| **HAdV** | HAdV-F | GCCACGGTGGGGTTTCTAAACTT | Hexon | [8] |
|  | HAdV-R | GCCCCAGTGGTCTTACATGCACAT | Hexon |  |
|  | HAdV-probe | Fam-TGCACCAGACCCGGGCTCAGGTACTCCGA-Tamra | Hexon |  |
|  |  |  |  |  |
| **HBoV** | HBoV-F | GCACAGCCACGTGACGAA | NP1 | [9] |
|  | HBoV-probe | Fam-TGAGCTCAGGGAATATGAAAGACAAGCATCG-Tamra | NP1 |  |
|  | HBoV- R | TGGACTCCCTTTTCTTTTGTAGGA | NP1 |  |
|  |  |  |  |  |
| **β-actin** | β-actin F | CCCAGCCATGTACGTTGCTA | β-actin | [10] |
|  | β-actin R | TCACCGGAGTCCATCACGAT | β-actin |  |
|  | β-actin-probe | Fam-ACGCCTCTGGCCGTACCACTGG-Tamra | β-actin |  |
|  |  |  |  |  |
| **RNAseP** | RNAseP for | AGATTTGGACCTGCGAGCG | RNAse P | [6] |
|  | RNAsePrev | GAGCGGCTGTCTCCACAAGT | RNAse P |  |
|  | RNAseP-prob | Fam-TTCTGACCTGAAGGCTCTGCGCG-BHQ1 | RNAse P |  |

**REFERENCES**

1. Hu A, Colella M, Tam JS, Rappaport R, Cheng SM (2003) Simultaneous detection, subgrouping, and quantitation of respiratory syncytial virus A and B by real-time PCR. J Clin Microbiol 41: 149-154.

2. Bouscambert-Duchamp M, Lina B, Trompette A, Moret H, Motte J, et al. (2005) Detection of human metapneumovirus RNA sequences in nasopharyngeal aspirates of young French children with acute bronchiolitis by real-time reverse transcriptase PCR and phylogenetic analysis. J Clin Microbiol 43: 1411-1414.

3. Deffernez C, Wunderli W, Thomas Y, Yerly S, Perrin L, et al. (2004) Amplicon sequencing and improved detection of human rhinovirus in respiratory samples. J Clin Microbiol 42: 3212-3218.

4. Kuypers J, Martin ET, Heugel J, Wright N, Morrow R, et al. (2007) Clinical disease in children associated with newly described coronavirus subtypes. Pediatrics 119: e70-76.

5. Garbino J, Gerbase MW, Wunderli W, Deffernez C, Thomas Y, et al. (2004) Lower respiratory viral illnesses: improved diagnosis by molecular methods and clinical impact. Am J Respir Crit Care Med 170: 1197-1203.

6. CDC (2009) Protocol of real time RT-PCR for influenza A. Available: http://www.who.int/csr/resources/publications/swineflu/realtimeptpcr/en/index.html. Accessed 22 Februaryt 2012

7. van Elden LJ, Nijhuis M, Schipper P, Schuurman R, van Loon AM (2001) Simultaneous detection of influenza viruses A and B using real-time quantitative PCR. J Clin Microbiol 39: 196-200.

8. Heim A, Ebnet C, Harste G, Pring-Akerblom P (2003) Rapid and quantitative detection of human adenovirus DNA by real-time PCR. J Med Virol 70: 228-239.

9. Neske F, Blessing K, Tollmann F, Schubert J, Rethwilm A, et al. (2007) Real-time PCR for diagnosis of human bocavirus infections and phylogenetic analysis. J Clin Microbiol 45: 2116-2122.

10. Nystrom K, Biller M, Grahn A, Lindh M, Larson G, et al. (2004) Real time PCR for monitoring regulation of host gene expression in herpes simplex virus type 1-infected human diploid cells. J Virol Methods 118: 83-94.
